# 1382. The Lesser Known Treponemal Infections: A Review Of Non-Syphilitic Cases

**DOI:** 10.1093/ofid/ofad500.1219

**Published:** 2023-11-27

**Authors:** Ashima Gupta, Saarthak Malhotra, Aasa Deepika Kuditipudi, Reema Charles, Tahsin Farid, Raghavendra Tirupathi, Barath Prashanth Sivasubramanian

**Affiliations:** Dr. Punjabrao dekhmukh memorial medical college, chicago, Illinois; Hamdard Institute of Medical Sciences and Research and HAHC Hospital, Olney, Maryland; DR Pinnamaneni Siddhartha Institute of Medical Sciences and Research Foundation, vijayawada, Andhra Pradesh, India; FDA, Silver Spring, Maryland; US Food & Drug Administration, Richmond, Texas; Keystone Health, Chambersberg, Pennsylvania; University of Texas Health San Antonio, San Antonio, Texas

## Abstract

**Background:**

Nonvenereal treponemal (NVT) infections (Bejel, Yaws, and Pinta) are historically a major health concern in developing countries^1^ (Figure 1), but they are increasingly ignored. NVTs can lead to severe complications. Our aim was to highlight disease characteristics, treatments, and outcomes, and to identify drug repurposing by curating all cases of NVTs in the literature in CURE ID^4^, a platform that catalogs real-world data on drug repurposing for difficult-to-treat infectious diseases.

Geographical distribution of Contracted Nonvenereal Treponemal Infection

**Figure:**
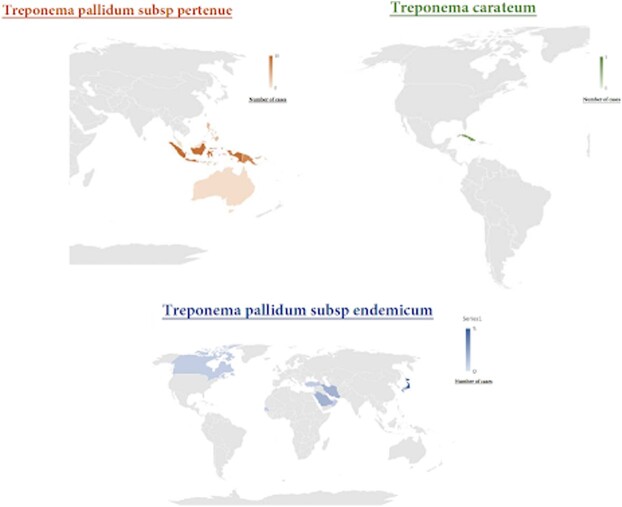

Yaws caused by Treponema pallidum subsp pertenue was primarily prevalent in Southeast Asia, particularly Indonesia, and Papua New Guinea. In contrast, Treponema pallidum subsp endemicum, which causes Bejel, was commonly found in Japan. Treponema carateum, the causative agent of Pinta, was limited to Cuba.

Geographical distribution of treated Nonvenereal Treponemal Infection

**Figure:**
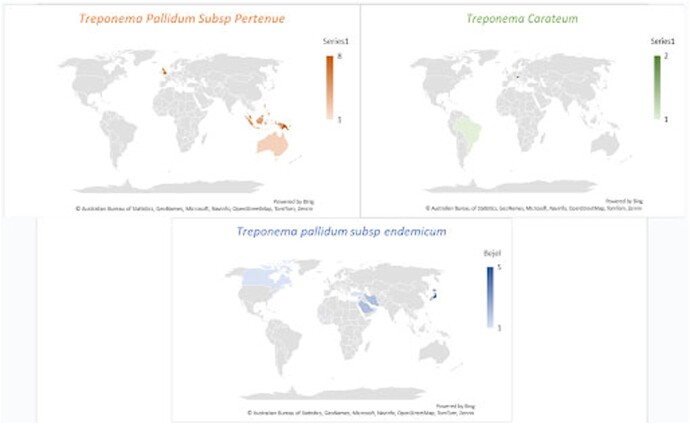

Treponema Pallidum Subsp Pertenue (Yaws) was mainly treated in Southeast Asia, Indonesia, and Papua New Guinea, as well as in England following identification after migration. Treponema pallidum subsp endemicum (Bejel), was typically treated in Japan. Treponema Carateum (Pinta), was managed in South America.

**Methods:**

We identified all case reports on NVTs from PubMed and Embase databases using relevant keywords, MeSH, and Emtree terms. Rayyan.ai^3^ was used to screen articles following a PRISMA protocol (Figure 3). All included articles were uploaded using the CURE ID case report form. De-identified, standardized data on NVTs from CURE ID was then analyzed.

PRISMA protocol for Nonvenereal Treponemal Infections

**Figure:**
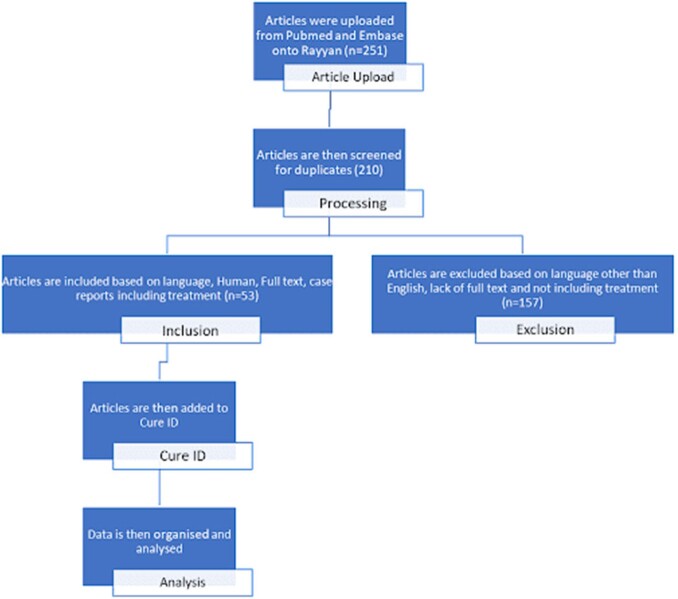

Out of 251 cases, identified on Pubmed and Embase, 53 cases met the inclusion criteria and were included in the analysis. We included cases published in English and those where antibiotics were used. All the case reports were added to CURE ID, organized, and analyzed.

The CURE ID Home Page

**Figure:**
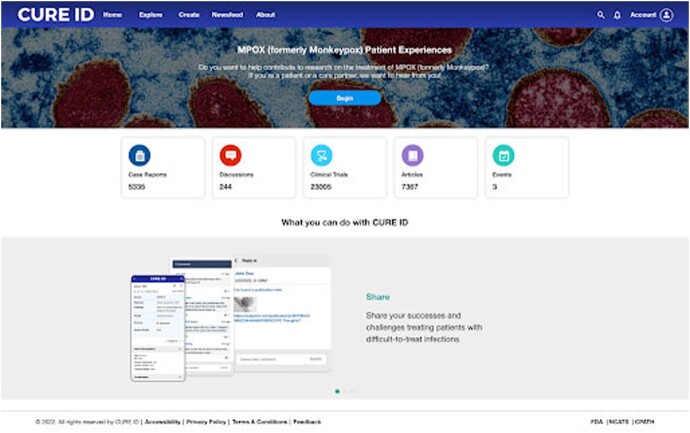

CURE ID captures treatment outcomes when drugs are used to treat new diseases or are used in other new ways. Through CURE ID’s ability to obtain and organize this information received directly from healthcare providers, patients, caregivers, electronic health records, and published case reports, promising new therapeutic uses can be identified for formal study by institutions involved in drug development, and unhelpful or harmful uses can potentially be avoided.

**Results:**

53 cases of NVTs were included: Yaws (37), Bejel (13), and Pinta (3). Most originated in developing countries (Figure 1), but many were treated in OECD countries (figure 2). Yaws predominantly occurred in children (1-10 years), while Bejel and Pinta were evenly distributed across age groups. Table 1 breaks down the NVTs according to gender and common sites of infection and Table 2 according to gender and symptoms. Diagnosis was often by clinical assessment (49/53) and serology (46/53) (Table 3). Penicillin was the most frequently used antibiotic, followed by azithromycin (Table 4).

Common sites of infection caused by Nonvenereal Treponemal infections

**Figure:**
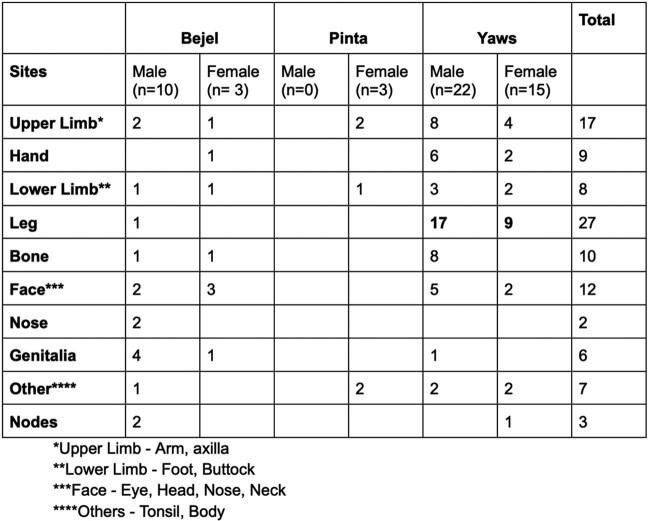

The presentations of Bejel and Yaws varied. In Bejel, men (4/10) primarily experienced genital involvement, and women (3/3) exhibited frequent facial involvement. In Yaws, both genders commonly experienced leg involvement. Meanwhile, Pinta usually presented with skin rashes/lesions all over the body in women and was not reported in men.

Common symptoms caused by Nonvenereal Treponemal infections

**Figure:**
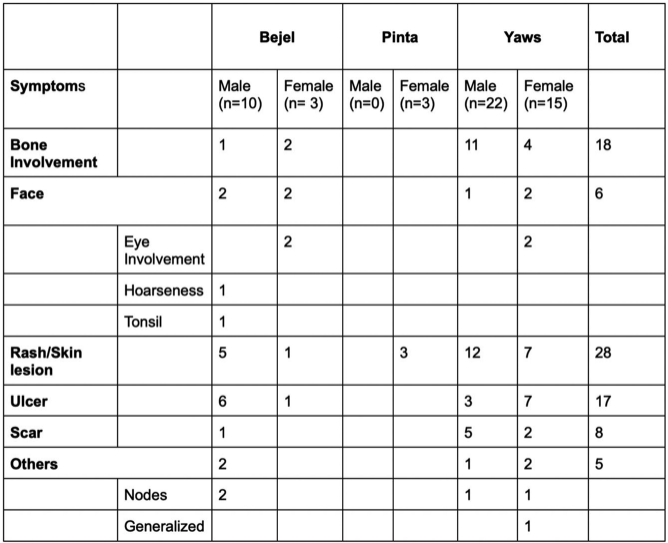

In the 37 cases of Yaws, skin rash/lesion (19/37) was the predominant presentation, followed by bone involvement (15/37) and ulcer (10/37). Bejel, on the other hand, commonly manifested with ulcers (7/13) and skin rash/lesion (6/13). In Pinta all three cases presented with skin rash/lesion.

Investigations leading to the diagnosis

**Figure:**
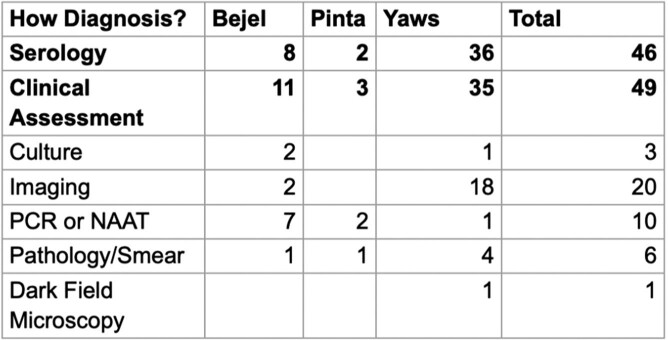

Clinical assessment (49/53) and serology (46/53 cases) were commonly used diagnostic methods for Nonvenereal Treponemal infections.

**Conclusion:**

Most cases originated in developing countries such as Indonesia (Figure 1), but many were only treated when patients emigrated to OECD countries (Figure 2). Interestingly, all cases of Bejel in females (3/3) had face or eye involvement, while symptoms were more varied for males (Table 2). This may be coincidental, but further investigation could identify gender-based differences in disease presentation. Most cases of NVT (52/53) either improved or were cured when treated (Table 5). Penicillin is FDA approved for NVTs and was the most often used drug (44/53). The successful use of azithromycin in some cases (7) indicates its potential as a second-line agent if resistance to penicillin arises. This project highlights the utility of CURE ID in using real-world data to identify signals of drug repurposing where traditional efficacy trials cannot be conducted.

Antibiotics used to treat Nonvenereal Treponemal infections

**Figure:**
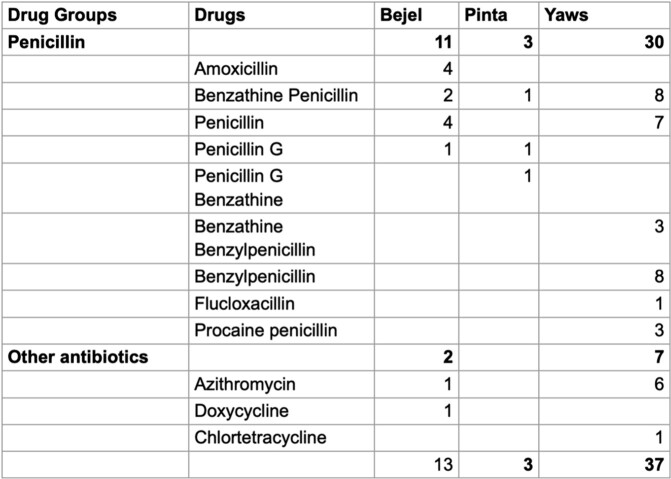

Penicillin was the most frequently used antibiotic class in 11/13 (84.61%), 30/37 (81.08%), and 3/3 (100%) of Bejel, Yaws, and Pinta respectively. Among other antibiotics, azithromycin was prescribed in 1/11 in Bejel and 6/37 in Yaws.

Outcomes of Nonvenereal Treponemal infections

**Figure:**
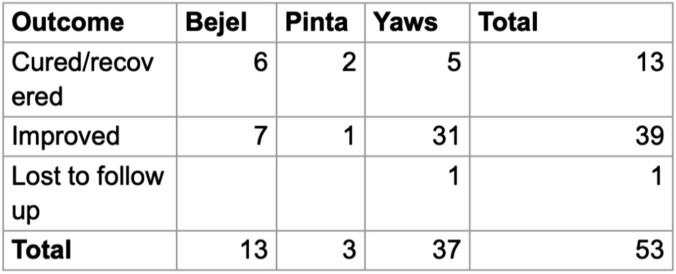

The majority of Nonvenereal Treponemal infections (52/53) either showed improvement or were cured.

**Disclosures:**

**All Authors**: No reported disclosures

